# Delay and Probability Discounting of Sexual and Monetary Outcomes in Individuals with Cocaine Use Disorders and Matched Controls

**DOI:** 10.1371/journal.pone.0128641

**Published:** 2015-05-27

**Authors:** Matthew W. Johnson, Patrick S. Johnson, Evan S. Herrmann, Mary M. Sweeney

**Affiliations:** Behavioral Pharmacology Research Unit, Department of Psychiatry and Behavioral Sciences, Johns Hopkins University School of Medicine, Baltimore, Maryland, United States of America; Institutes for Behavior Resources and Johns Hopkins University School of Medicine, UNITED STATES

## Abstract

Individuals with cocaine use disorders are disproportionately affected by HIV/AIDS, partly due to higher rates of unprotected sex. Recent research suggests delay discounting of condom use is a factor in sexual HIV risk. Delay discounting is a behavioral economic concept describing how delaying an event reduces that event’s value or impact on behavior. Probability discounting is a related concept describing how the uncertainty of an event decreases its impact on behavior. Individuals with cocaine use disorders (*n* = 23) and matched non-cocaine-using controls (*n* = 24) were compared in decision-making tasks involving hypothetical outcomes: delay discounting of condom-protected sex (Sexual Delay Discounting Task), delay discounting of money, the effect of sexually transmitted infection (STI) risk on likelihood of condom use (Sexual Probability Discounting Task), and probability discounting of money. The Cocaine group discounted delayed condom-protected sex (i.e., were more likely to have unprotected sex vs. wait for a condom) significantly more than controls in two of four Sexual Delay Discounting Task partner conditions. The Cocaine group also discounted delayed money (i.e., preferred smaller immediate amounts over larger delayed amounts) significantly more than controls. In the Sexual Probability Discounting Task, both groups showed sensitivity to STI risk, however the groups did not differ. The Cocaine group did not consistently discount probabilistic money more or less than controls. Steeper discounting of delayed, but not probabilistic, sexual outcomes may contribute to greater rates of sexual HIV risk among individuals with cocaine use disorders. Probability discounting of sexual outcomes may contribute to risk of unprotected sex in both groups. Correlations showed sexual and monetary results were unrelated, for both delay and probability discounting. The results highlight the importance of studying specific behavioral processes (e.g., delay and probability discounting) with respect to specific outcomes (e.g., monetary and sexual) to understand decision making in problematic behavior.

## Introduction

Approximately 1.5 million people in the U.S. have used cocaine within the past month [1]. HIV prevalence among individuals who use cocaine (4–22%) [2–6] is many times higher than the national average (0.4%) [7]. Only about ten percent of individuals who use cocaine inject [8–10], and HIV rates among injecting vs. non-injecting individuals who use cocaine are similar [2, 5, 11], suggesting that risky sexual behavior is the most prominent HIV transmission vector among individuals who use cocaine.

Most sexual HIV risk reduction interventions for individuals who use cocaine target HIV risk reduction knowledge (e.g., [12–14]) and condom use skills (e.g., [15]). Although these interventions increase knowledge and skills, meta-analyses have demonstrated less robust effectiveness in reducing risk behavior [16–17]. The observation that individuals who use cocaine continue to engage in risky sexual behavior despite knowledge/skills improvements prompts examination of other factors that may underlie risk behavior, including decision-making processes.

Delay discounting provides a useful framework for examining relations between decision making and risk behavior. Delay discounting is a concept from the field of behavioral economics describing how delaying an event reduces that event’s value or impact on behavior. This is shown, for example, by the observation that individuals typically prefer immediate over delayed rewards. Most delay discounting studies ask participants to make choices between receiving smaller amounts of money available immediately vs. larger amounts available after various delays. Steeper discounting of monetary rewards is related to cocaine use (e.g., [18–21]) and use of other substances [22], as well as a variety of non-drug-related problem behaviors, including pathological gambling [23–24], obesity [25–26], and failing to engage in preventive health behaviors [27–29]. However, choices between immediate and delayed outcomes involve a variety of reinforcers other than money. For example, in a casual sex scenario, one may prefer to use a condom because it decreases the risk of sexually transmitted infection (STI). However, if a condom is not readily available, the same person might prefer immediate unprotected sex over waiting to obtain a condom. In other words, the value of condom protection may be discounted due to delay.

The Sexual Delay Discounting Task (previously referred to as the “Sexual Discounting Task”) was developed to assess the influence of delay on choices related to condom use in casual sex scenarios. Studies using the task in individuals with cocaine use disorders [30–31] reported several findings. First, individuals with cocaine use disorders generally indicated that they would be less likely to use condoms as the delay to condom availability increased. Second, participants discounted condom-protected sex more steeply for partners with whom they most vs. least wanted to have sex, and for partners they judged least vs. most likely to have an STI. Third, steeper discounting of condom-protected sex was significantly associated with higher rates of self-reported sexual HIV risk behavior. Fourth, the Sexual Delay Discounting Task showed good 1-week test-retest reliability. Together, these findings suggest the Sexual Delay Discounting Task has both external and internal validity among individuals with cocaine use disorders.

Despite the reliability and validity of the Sexual Delay Discounting Task within individuals with cocaine use disorders, there are no reports comparing delay discounting of condom-protected sex between individuals with cocaine use disorders and those who do not use cocaine. A recent study using the Sexual Delay Discounting Task demonstrated that opioid-dependent women discounted delayed condom-protected sex and monetary rewards more steeply than non-drug-using control women, and that participants in both groups discounted in an orderly manner that was sensitive to partner characteristics [32]. Moreover, a recent study in 18–24 year old youth found increased delay discounting of condom-protected sex to be significantly associated with greater self-reported drug use [33]. These findings suggest that steeper discounting may be related to higher rates of sexual HIV risk in drug-using populations, but it is unknown whether steeper discounting is related to the higher rates of sexual HIV risk behavior observed specifically among individuals who use cocaine. It is worthwhile to examine the relationship between the discounting of sexual outcomes and cocaine use because individuals who use cocaine have shown higher rates of sexual risk behavior [34] and greater delay discounting of monetary rewards [35] than individuals who use heroin. The present study therefore compared discounting of delayed sexual and monetary outcomes between individuals with cocaine use disorders and matched non-cocaine-using controls.

Beyond delay, at least one additional factor that may influence condom use is the probability of contracting an STI with unprotected sex. Indeed, one reason condoms are ever preferred is likely because they decrease the probability of aversive outcomes (i.e., STIs or unwanted pregnancy). Therefore, we also compared probability discounting of sexual and monetary outcomes between the two groups. With methods analogous to delay discounting, probability discounting tasks systematically examine how uncertainty influences an event’s value or impact on behavior [36–37]. While delay and probability discounting are only weakly correlated, the two processes have shown independent associations with clinically relevant behavior (i.e., gambling) and show differential effects of experimental manipulations (i.e. reward magnitude manipulations show directionally opposite effects on delay and probability discounting). Therefore, delay and probability discounting likely represent separate behavioral processes (e.g., [38–41]). With respect to the influence of probability on sexual outcomes, previous studies using the Sexual Delay Discounting Task showed that a partner’s perceived likelihood of having an STI influenced delay discounting of condom use. However, these studies did not explicitly manipulate the probability of STI contraction. We developed the Sexual Probability Discounting Task to quantitatively examine how specified risk of STI contraction resulting from unprotected sex influences condom use. The reliably weak correlations observed in previous studies between delay and probability discounting of monetary rewards also prompted us to examine correlations between discounting in the Sexual Delay and Sexual Probability Discounting tasks.

## Materials and Methods

### Ethics Statement

Study procedures were approved by the Johns Hopkins Medicine Institutional Review Board 3 (Office for Human Research Protections Registration #00001656). The study was conducted according to the principles expressed in the Declaration of Helsinki. Written informed consent was obtained from the participants.

### Participants

Volunteers were recruited using flyers, Internet, newspaper, and radio advertisements, and word of mouth referral. Inclusion criteria for both the cocaine use disorder (Cocaine) and non-cocaine-using (Control) groups included being at least 18 years of age, having at least an 8^th^ grade reading level, and reporting having vaginal or anal intercourse with another person during their lifetime. Participants in the Cocaine group met Diagnostic and Statistical Manual of Mental Disorders (4^th^ edition, DSM-IV) criteria for cocaine abuse or dependence, whereas participants in the Control group reported no lifetime use of cocaine. Participants in both groups could meet criteria for abuse for drugs other than cocaine, but could not meet dependence criteria for other drugs (excluding nicotine and caffeine). Exclusion criteria for both groups included self-reported serious head trauma, dementia, significant cognitive impairment, or diagnosis of major psychiatric disorder besides substance abuse/dependence.

### Procedure

After an initial telephone screening assessing basic inclusion/exclusion criteria, initially qualified participants were scheduled for an in-person screening. If qualified, participants remained in the laboratory for approximately four hours to complete a variety of behavioral tasks. During the in-person screening, participants provided informed consent and a urine sample to test for drug use. Participants also completed a demographic questionnaire, a verbal intelligence assessment (Quick Test) [42], a reading comprehension assessment (Wide Range Achievement Test) [43], a lifetime drug use questionnaire, and a checklist to assess current and past drug abuse and dependence [44]. Occurrence and frequency of HIV risk behaviors in the past month were assessed using the HIV Risk-Taking Behavior Scale (HRBS) [45–46]. The HRBS is a psychometrically reliable and valid questionnaire featuring 11 items scored on a 6-point scale (scores of 0–5, with higher scores indicating higher risk) pertaining to injection drug use (6 items) and sexual risk behavior (5 items). Only scores on the sexual risk behavior subscale, which assessed participants’ number of sexual partners in the past month, frequency of condom use with regular and casual partners and when paid for sex, and frequency of anal sex, were compared between groups. Several personality measures and behavioral tasks were also obtained but are not relevant to the present analyses.

As in our previous sexual discounting studies [30–33], participants then used a computer to view 60 individually-presented color photographs of diverse, clothed people (30 male, 30 female) and were asked to select photographs of individuals that they would consider having casual sex with based on physical appearance. The photographs were assembled from a variety of publicly available online repositories to provide a range of physical appearances that would be conducive to the multiple hypothetical partner conditions described below. Before viewing photographs (and before subsequent sexual tasks), participants were instructed to pretend that they were not in a committed relationship. Next, participants identified from the subset of initially selected photographs the person they (1) most wanted to have sex with, (2) least wanted to have sex with, (3) judged was most likely to have an STI, and (4) judged was least likely to have an STI. A single photograph could be assigned to multiple partner conditions, but not for “most” and “least” categories within one dimension. Participants who selected fewer than two photographs (*n* = 2 potential Control group participants) were disqualified from further participation. Female participants who selected only photographs of females would have been disqualified from further participation because the risk of HIV infection from female-female sex is extremely low [47], although this criterion resulted in no exclusions for the current study. Participants who were disqualified from further participation were compensated $30.

Qualified participants were then trained on using a visual analog scale. Next, they completed the four discounting tasks described below, in addition to other decision-making tasks not relevant to the present analyses. Monetary tasks were administered before sexual tasks, and delay tasks were administered before probability tasks.

#### Sexual Delay Discounting Task

Delay discounting of condom-protected sex was assessed using a computerized version of the Sexual Delay Discounting Task [32]. At the beginning of each of the four partner conditions (presented in a pseudo-randomized order), the participant was shown the relevant photograph and instructed to imagine the person was interested in having sex now, that there was no chance of pregnancy, and that a condom was readily and immediately available. The participant indicated his/her likelihood of using a condom by clicking on a visual analog scale that ranged from “I will definitely have sex with this person without a condom” (0%) to “I will definitely have sex with this person with a condom” (100%). In subsequent trials, the participant was asked to rate his/her likelihood of waiting a given delay (ascending order; 1 hour, 3 hours, 6 hours, 1 day, 1 week, 1 month, and 3 months) to have sex with a condom. The visual analog scales for these trials ranged from “I will definitely have sex with this person now without a condom” (0%) to “I will definitely wait [delay] to have sex with this person with a condom” (100%). In the event that a photograph was assigned to multiple partner conditions, the participant completed the 8-trial series only once for that photograph.

#### Monetary Delay Discounting Task

Delay discounting of hypothetical money was assessed using a computerized task used previously [19–20, 48–53]. Participants made choices between smaller amounts of money delivered immediately vs. a larger amount ($100) delivered after a delay. Unlike the Sexual Delay Discounting Task, which compared groups with respect to decisions involving each of four hypothetical partners, for the Monetary Delay Discounting only a single condition (i.e., reinforcer magnitude) was evaluated for group differences. Participants were instructed to treat choices as if the outcomes were real and that they should take their financial circumstances into account when making their choices. Based on the pattern of a participant’s choices, the task algorithm calculated an indifference point (i.e., a smaller amount of money subjectively equivalent to delayed $100) at each of 7 delays: 1 day, 1 week, 1 month, 6 months, 1 year, 5 years, and 25 years (see [49] and [54] for a description of the algorithm used to determine indifference points). Although some delays were common to both discounting tasks (i.e., 1 day, 1 week, and 1 month), the standard range of delays typically assessed in the Monetary Delay Discounting Task exceeded that of the Sexual Delay Discounting Task. The order in which delays were assessed (ascending or descending) was randomly determined.

#### Sexual Probability Discounting Task

In this task, participants were asked to imagine in each decision that having sex with a photographed individual was associated with a specified risk of contracting an STI. We administered the task only for the “most want to have sex with” and “least want to have sex with” partner conditions to avoid explicitly confounding our experimental manipulation of risk with the perceived risk of partners. Presentation order was identical to that of the Sexual Delay Discounting Task. At the beginning of each partner condition, a research assistant placed a printed copy of the relevant photograph (21.59 cm x 27.94 cm) on the desk in front of the participant and reminded her or him to imagine the person was interested in having sex now, and that there was no chance of pregnancy. For the first trial, the research assistant read text shown to the participant specifying that if he/she did not use a condom, then there was a 1 in 1 (100%) chance of contracting an STI from the photographed individual. A visual analog scale located below the prompt ranged from “I will definitely have sex with this person without a condom” to “I will definitely have sex with this person with a condom” for this and all other probability trials (see [55] for use of a visual analog scale to assess probability discounting). No trials involved delays. In all trials, risk was described both as odds in favor and percent chance of contracting an STI. Other risk values assessed (in descending order) were 1 in 3 (33%), 1 in 13 (8%), 1 in 100 (1%), 1 in 400 (0.25%), 1 in 700 (0.14%), 1 in 2,000 (0.05%), and 1 in 10,000 (0.01%).

#### Monetary Probability Discounting Task

Probability discounting of hypothetical money ($100) was assessed using a computerized task used in a previous study [52]. Choices were between smaller amounts of money delivered immediately with 100% certainty vs. a larger amount ($100) delivered immediately, but with a specified probability of delivery. Indifference points were obtained at each of 7 probabilities of receiving $100: 99%, 90%, 75%, 50%, 25%, 10%, and 1%. Hypothetical rewards instructions and the choice-adjustment algorithm were identical to the Monetary Delay Discounting Task. The order in which probability values were assessed (ascending or descending) was matched to the Monetary Delay Discounting Task for each participant.

#### HIV testing

Upon completion of all experimental tasks, a 3.5 cc blood specimen was collected and sent to a commercial laboratory (Quest Diagnostics Incorporated, Baltimore, MD) for HIV-1/HIV-2 antibody testing. Immediately after the blood draw, participants were discharged and compensated $75 for study completion.

#### Evaluation of group characteristics

Group characteristics were compared using independent-samples *t*-tests for continuous variables and Fisher’s exact tests for categorical variables. Groups were matched (i.e., no significant or trend-level differences, *t*s[45] ≤ 1.62, *p*s ≥. 11) on the following characteristics: age, sex, race, ethnicity, marital status, educational attainment, monthly income, Quick Test score, and cigarettes per day.

#### Orderliness of discounting data

Likelihood values from the sexual tasks were calculated as a proportion of the total length of the visual analog scale and indifference points from the monetary tasks were expressed as a proportion of $100. Nonsystematic discounting data were identified according to criteria adapted from a previously established algorithm [56–57]. For the sexual tasks, two criteria were used to identify nonsystematic data. First, starting with the second delay value (1 hour) or probability value (33%), the likelihood of condom use at a given delay or probability could not exceed the immediately preceding likelihood by more than 0.2. Second, the likelihood of condom use at the longest delay (3 months) or smallest probability (0.01%) could not exceed the likelihood when a condom was immediately available or the risk of contracting an STI was 100% by more than 0.1. For the monetary tasks, the analogous two criteria were applied as well as an additional criterion, which specified that the final indifference point could not be greater than 0.9. Participants whose data violated one or more of the aforementioned criteria in a condition were excluded in analyses of that condition.

#### Discounting data analysis

Groups were compared using extra sums-of-squares *F* tests analyzing all individual participant data as previously described [20, 58] (GraphPad Prism version 6.05 for Windows, GraphPad Software, La Jolla, CA). For delay tasks, these analyses regressed proportion likelihood of condom use (sexual tasks) or indifference points (monetary tasks) against delay (hours). For probability tasks, these measures were regressed against odds against, which were calculated as (1/*p*)-1, wherein *p* is the probability in favor of an event’s occurrence [37]. Nonlinear regressions were performed using a two-parameter hyperbolic discounting equation [59–61]: Proportion likelihood or indifference point = 1/(1+*r*X)^*s*^, wherein X is the task-specific independent variable (i.e., either hours or odds against), *r* is a parameter proportional to discounting rate, and *s* is a parameter describing the nonlinear scaling of the dependent variable (monetary amount or likelihood of condom use) and delay or odds against. The free parameters *r* and *s* were unconstrained in regression analyses. The *F* tests compared the difference in nonlinear regression model error when free parameters were shared (i.e., one curve best-fit to all discounting data collapsed across groups) vs. when free parameters were unshared (i.e., a separate best-fit for each group). A significant *p* value indicates significantly less error when model parameters are unshared, indicating that the groups differ.

In addition to analyzing raw group likelihood data from the Sexual Delay Discounting and Sexual Probability Discounting tasks, we also compared 0-delay (and 100%-probability) likelihood values, which were non-normally distributed, between Cocaine and Control groups using Mann-Whitney *U* tests.

For the Sexual Delay Discounting Task we also isolated the effect of delay on likelihood of waiting to use delayed condoms (i.e., discounting) from differences in preference for using immediately available condoms (0-delay). Specifically, individual participant raw likelihood values for each condition were standardized by dividing each non-zero delay trial likelihood value by its respective 0-delay trial value. In the event that a standardized likelihood value exceeded 1 (i.e., a non-zero delay trial likelihood exceeded its respective 0-delay trial value), the value was replaced with a value of 1. Participants who reported zero likelihood of using an immediately available condom were excluded from standardized data analyses because the relative effect of delay on reward value is undefined if the initial value is zero. An identical procedure was employed to standardize the Sexual Probability Discounting Task data with respect to 100%-probability trial data. Extra sums-of-squares *F* tests were used to compare standardized discounting data between Cocaine and Control groups in each condition.

Correlations among all discounting measures (using standardized likelihood in the sexual tasks) were conducted using an area-under-the-curve (AUC; [62]) metric. Spearman’s rank-order correlations were calculated because AUC values were non-normally distributed. The criterion for significance in all tests was *p* <. 05.

## Results

### Sample characteristics

Table 1 presents characteristics of the Cocaine (*n* = 23) and Control (*n* = 24) groups. There were no significant differences between the groups with the exception of substance use, self-reported sexual risk behavior, and HIV variables. All 23 participants in the Cocaine group met criteria for cocaine abuse, and 20 participants also met criteria for cocaine dependence. Eighteen participants (78%) reported inhalation (smoking) as their preferred route of cocaine administration, and 4 (17%) and 1 (4%) participants preferred intranasal (snorting) and intravenous routes, respectively. Compared to the Control group, the Cocaine group reported significantly greater alcohol and cannabis use (number of past-year users; days used per month), and included significantly more participants meeting diagnostic abuse criteria for these two drugs. There were no significant group differences in measures of opioid and cigarette use. Participants in the Cocaine group also had significantly higher HRBS sexual risk subscale scores, and had a greater proportion of HIV-positive participants, than the Control group. Five of six Cocaine group participants with HIV knew they were HIV-positive prior to participating in the study and completing discounting assessments. Excluding these five HIV-positive participants did not alter whether 13 of 14 between-groups comparisons of discounting data met significance or not (the exception being a significant between-group difference in the standardized analysis of the “least likely to have an STI” partner condition, one that was not significant in the full sample). We therefore we report results from the full sample. In preparation for the sexual discounting tasks, 13 men in the Control group and 11 men in the Cocaine group selected exclusively female partners, while 7 women in the Control group and 10 women in the Cocaine group selected exclusively male partners. One man in the Control group and 2 men in the Cocaine group selected exclusively male partners, and 1 man in the Control group selected both male and female partners. Two women in the Control group selected both male and female partners.

**Table 1:** Characteristics of Cocaine and Control groups.

Characteristic	Cocaine (*n* = 23)	Control (*n* = 24)	Test statistic	*p* value
Demographics				
Age in years, mean (SD)	46.3 (10.9)	40.0 (15.3)	*t*(45) = 1.62	.11
Sex, count (%)				
Male	13 (57)	15 (63)	-	.77
Female	10 (43)	9 (38)		
Race, count (%) ^a^				
African-American/black	14 (61)	17 (71)	-	.76
Caucasian/white	8 (35)	7 (29)		
More than one race ^b^	1 (4)	0 (0)		
Marital status, count (%)				
Non-married (single/separated/divorced/widowed)	20 (87)	21 (88)	-	1.0
Married	3 (13)	3 (13)		
Education, years, mean (SD)	13.1 (1.7)	13.8 (1.6)	*t*(45) = 1.49	.14
Monthly income, US $, mean (SD)	1186 (826)	1369 (1222)	*t*(45) = 0.59	.55
Quick Test intelligence score ^c^	43.2 (3.4)	41.6 (4.1)	*t*(45) = 1.44	.16
Substance use				
Cocaine				
Number reporting use in past year (%)	23 (100)	-		
Frequency of use, days/month, mean (SD)	16.0 (9.1)	-		
Number meeting DSM-IV criteria for current abuse (%)	23 (100)	-		
Number meeting DSM-IV criteria for current dependence (%)	20 (87)	-		
Alcohol				
Number reporting use in past year (%)	22 (96)	17 (71)	-	.048
Frequency of use, days/month, mean (SD)	10.2 (10.4)	1.5 (3.3)	*t*(45) = 3.90	<.0001
Number meeting DSM-IV criteria for current abuse (%)	7 (30)	1 (4)	-	.02
Cannabis				
Number reporting use in past year (%)	16 (70)	6 (25)	-	<.01
Frequency of use, days/month, mean (SD)	5.2 (9.9)	2.0 (7.0)	*t*(45) = 1.30	.20
Number meeting DSM-IV criteria for current abuse (%)	5 (22)	0 (0)	-	.02
Opioids				
Number reporting use in past year (%)	6 (26)	2 (8)	-	.14
Frequency of use, days/month, mean (SD)	1.0 (3.2)	0.007 (0.02)	*t*(45) = 1.53	.13
Number meeting DSM-IV criteria for current abuse (%)	1 (4)	0 (0)	-	.49
Cigarettes smoked per day, mean (SD)	6.9 (5.4)	4.3 (7.6)	*t*(45) = 1.39	.17
Sexual risk behavior and HIV and HCV status				
HRBS Sexual Risk Subscale Score, mean (SD)	9.5 (5.0)	4.0 (4.5)	*t*(45) = 3.98	<.0001
HIV-positive, count (%) ^d^	6 (26)	0 (0)	-	<.01
HCV-positive, count (%) ^e^	2 (9)	0 (0)	-	.23

*Note*: Percentages may not add to 100 due to rounding error.

^a^ All participants identified as Non-Hispanic.

^b^ Race categories were evaluated as Caucasian/white vs. other to test distributions across Cocaine and Control groups.

^c^ Max = 50; adult norms: *M* = 41.4, SD = 6.0 [42].

^d^ Participants who tested positive were provided HIV counseling (including contact with follow-up care) by our nursing staff.

^e^ Assessed via questionnaire during in-person screening interview.

Sexual discounting data from partner conditions in which female participants selected a female partner (*n* = 4; 1 partner condition for 1 participant and 3 partner conditions for 1 participant) were excluded prior to analysis because the risk of female-female HIV transmission via sexual behavior is extremely low [47].

### Orderliness of data

Across all four partner conditions of the Sexual Delay Discounting Task, 77% and 85% of discounting functions were systematic for Cocaine and Control groups, respectively. Similarly, 87% (Cocaine) and 88% (Control) of Monetary Delay Discounting Task functions were systematic.

Across both partner conditions of the Sexual Probability Discounting Task, 98% (Cocaine) and 100% (Control) of discounting functions were systematic and 100% (Cocaine) and 96% (Control) of Monetary Probability Discounting Task functions were systematic.

Excluding nonsystematic data and data that could not be standardized (because of a zero initial likelihood of condom use) resulted in *n* that differed somewhat from analysis to analysis, however, these exclusions did not alter whether any group difference reported in Table 1 reached significance or a trend toward significance.

### Between-group comparisons of discounting data

#### Sexual Delay Discounting\

Reported likelihood of waiting to use a condom decreased as a function of delay to condom availability in all four Sexual Delay Discounting Task partner conditions for both groups. Significant group differences (i.e., lower likelihood of waiting to use a condom among Cocaine participants than among Control participants) were observed in two partner conditions: the “most want to have sex with” partner condition [Cocaine *n* = 19, Control *n* = 19, *F*(2, 300) = 5.81, *p* <. 01], and the “least want to have sex with” partner condition [Cocaine *n* = 13, Control *n* = 20, *F*(2, 260) = 6.38, *p* <. 01].

**Fig 1:**
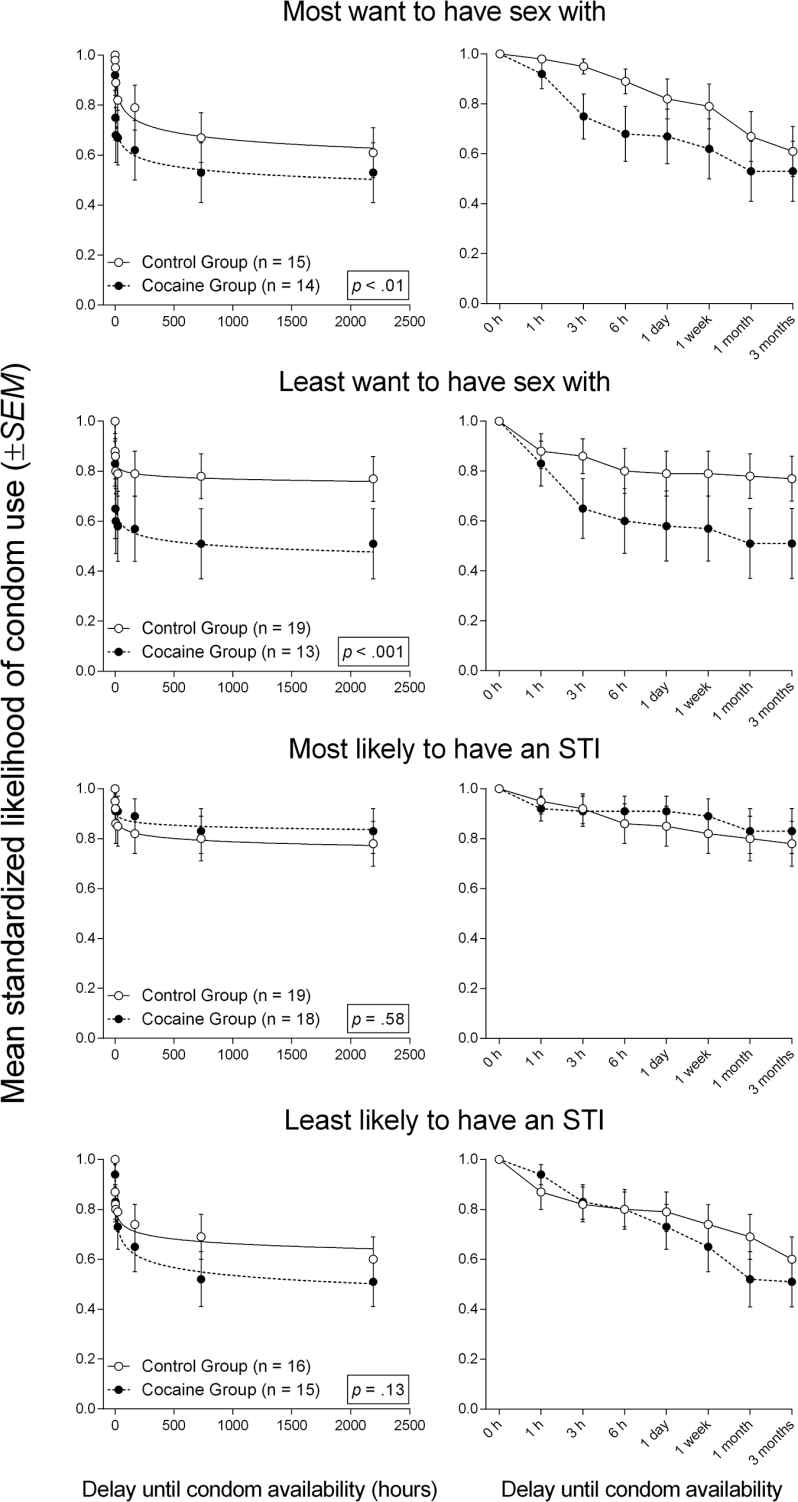
Best-fit curves to mean standardized likelihood of condom use in the Sexual Delay Discounting Task. Left column: Best-fit curves to mean standardized likelihood of condom use (proportion of visual analog scale) in each of the Sexual Delay Discounting Task partner conditions in the Control and Cocaine groups. Right column: Data from left column with delay to condom availability expressed ordinally on the x-axis. Error bars represent ±*SEM*.

Fig 1 (left column) shows best-fit curves to mean standardized likelihood of condom use in Cocaine and Control groups within each partner condition of the Sexual Delay Discounting Task. The right column of Fig 1 displays these same data, except with delay to condom availability expressed ordinally to facilitate visual inspection at short delays. When data were standardized to isolate the effect of delay, participants in the Cocaine group discounted significantly more steeply than those in the Control group in two of four partner conditions: the “most want to have sex with” partner condition [Cocaine *n* = 14, Control *n* = 15, *F*(2, 228) = 5.51, *p* <. 01], and the “least want to have sex with” partner condition [Cocaine *n* = 13, Control *n* = 19, *F*(2, 252) = 8.56, *p* <. 001]. Discounting did not differ significantly between Cocaine and Control groups with the “most likely to have an STI” partner condition [Cocaine *n* = 18, Control *n* = 19, *F*(2, 292) = 0.55, *p* =. 58], or the “least likely to have an STI” partner condition [Cocaine *n* = 15, Control *n* = 16, *F*(2, 244) = 2.02, *p* =. 13]. No significant group differences in likelihood of using an immediately available condom were observed (*U*s ≥ 116.5, *p*s ≥. 25).

#### Monetary Delay Discounting

**Fig 2:**
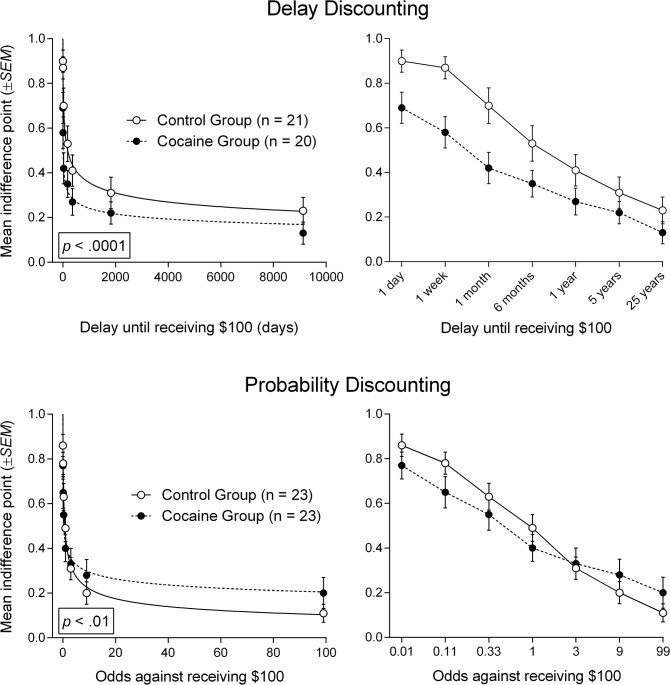
Best-fit curves to mean indifference points from monetary discounting tasks. Top row: Best-fit curves to mean indifference points (proportion of $100) from the Monetary Delay Discounting Task (left graph; right graph shows data with delay expressed ordinally). Bottom row: Best-fit curves to mean indifference points (proportion of $100) from the Monetary Probability Discounting Task (left graph; right graph shows data with odds against expressed ordinally). Error bars represent ±*SEM*.

The top row of Fig 2 shows best-fit curves to delay discounting data for hypothetical money (left graph; right graph shows data with delays expressed ordinally). Cocaine participants (*n* = 20) discounted delayed $100 significantly more steeply than Control participants [*n* = 21, *F*(2, 283) = 18.29, *p* <. 0001].

#### Sexual Probability Discounting

Reported likelihood of using an immediately available condom decreased with increased odds against contracting an STI for both partner conditions in both groups. However, no significant differences in discounting between Cocaine and Control groups were detected in unstandardized likelihood of condom use in the Sexual Probability Discounting Task for either the “most want to have sex with” partner condition [Cocaine *n* = 23, Control *n* = 24, *F*(2, 372) = 2.30, *p* =. 10] or for the “least want to have sex with” partner condition [Cocaine *n* = 22, Control *n* = 23, *F*(2, 356) = 1.88, *p* =. 16].

**Fig 3:**
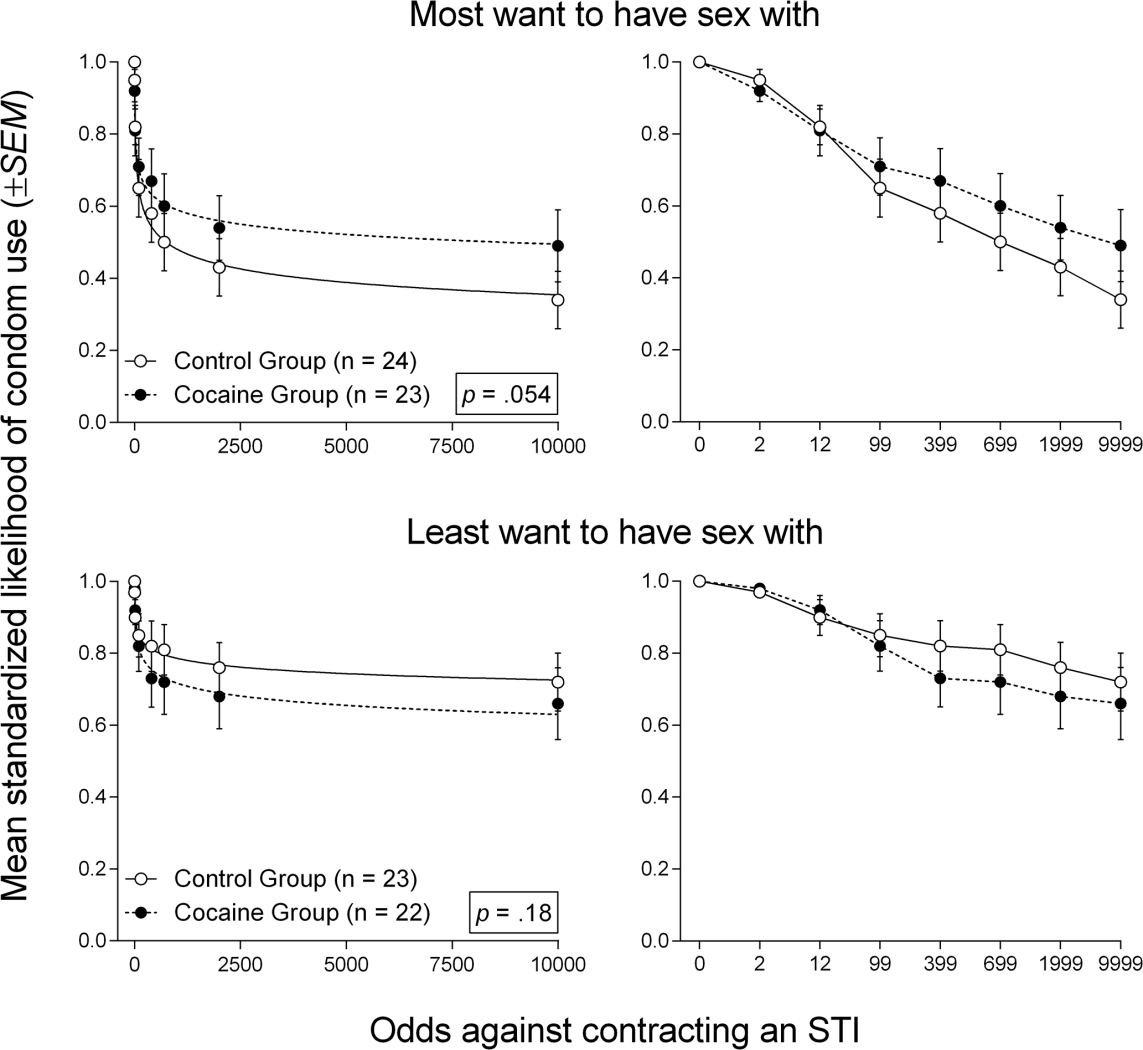
Best-fit curves to mean standardized likelihood of condom use in the Sexual Probability Discounting Task. Left column: Best-fit curves to mean standardized likelihood of condom use (proportion of visual analog scale) in each of the Sexual Probability Discounting Task partner conditions in the Cocaine and Control groups. Right column: Data from left column with odds against STI contraction expressed ordinally on the x-axis. Error bars represent ±*SEM*.

Fig3 (left column) shows best-fit curves to mean standardized likelihood of condom use in Cocaine and Control groups within each partner condition of the Sexual Probability Discounting Task. The right column of Fig 3 displays these same data, except with odds against contracting an STI expressed ordinally. Analyses of standardized data suggested a trend for those in the Cocaine group to discount condom-protected sex less steeply as STI risk decreased for the “most want to have sex with” partner [Cocaine *n* = 23, Control *n* = 24, *F*(2, 372) = 2.95, *p* =. 054], with no discounting difference for the “least want to have sex with” partner [Cocaine *n* = 22, Control *n* = 23, *F*(2, 356) = 1.72, *p* =. 18]. The groups also did not differ statistically with respect to likelihood of condom use when the risk of contracting an STI was 100% (*U*s ≥ 226, *p*s ≥. 23).

#### Monetary Probability Discounting

The bottom row of Fig 2 shows best-fit curves to probability discounting data for hypothetical money (left graph; right graph shows data with odds against expressed ordinally). Cocaine (*n* = 23) and Control (*n* = 23) group participants showed a significantly different pattern of discounting for a probabilistic $100 reward [*F*(2, 318) = 5.24, *p* <. 01]. However, the bottom right panel of Fig 2 shows that one group did not consistently discount to a greater or lesser extent than the other group. At lower odds against receiving $100 (i.e., 0.01, 0.11, 0.33, 1), mean indifference points for the Control group tended to be higher than mean indifference points for the Cocaine group, whereas at higher odds against receiving $100 (i.e., 9, 99), the Cocaine group tended to be higher than the Control group.

### Correlations Among Discounting Tasks

Table 2 shows Spearman rank correlations for sexual (using standardized likelihood) and monetary discounting tasks. Upon elimination of nonsystematic discounting data sets and sexual data sets showing zero likelihood of condom use under 0-delay or 100% probability of STI conditions, *n* ranged from 22 to 46 across these correlations. Within the Sexual Delay Discounting Task, discounting measures among partner conditions were positively and significantly correlated in 4 of 6 instances. Similarly, within the Sexual Probability Discounting Task, discounting between the two partner conditions was positively and significantly correlated. The Sexual Delay Discounting Task and the Sexual Probability Discounting Task were significantly and positively correlated in 7 of 8 partner conditions. Conversely, the Monetary Delay Discounting Task and Monetary Probability Discounting Task were not significantly correlated. The Monetary Delay Discounting Task was not significantly correlated with the Sexual Delay Discounting Task for any of the 4 partner conditions. Similarly, the Monetary Probability Discounting Task was not significantly correlated with either partner condition in the Sexual Probability Discounting Task. Finally, although the Monetary Probability Discounting Task was significantly and positively correlated with the Sexual Delay Discounting Task for 2 of the 4 partner conditions, no significant relation was found between the Monetary Delay Discounting Task and either partner condition in the Sexual Probability Discounting Task.

**Table 2:** Spearman rank correlation matrices for sexual and monetary discounting tasks.

Discounting Condition/Task	1	2	3	4	5	6	7
Sexual Delay Discounting Task							
1.Most want to have sex with	-						
2.Least want to have sex with	.26	-					
	(*n* = 22)						
3.Most likely to have an STI	.33	**.57**	-				
	(*n* = 25)	(*n* = 29)					
43Least likely to have an STI	**.62**	**.57**	**.48**	-			
	(*n* = 25)	(*n* = 25)	(*n* = 26)				
5.Monetary Delay Discounting Task	-.10	.08	.16	.00	-		
	(*n* = 24)	(*n* = 24)	(*n* = 31)	(*n* = 24)			
Sexual Probability Discounting Task							
6.Most want to have sex with	**.64**	**.46**	**.47**	**.60**	.05	-	
	(*n* = 29)	(*n* = 32)	(*n* = 37)	(*n* = 31)	(*n* = 37)		
7.Least want to have sex with	.21	**.55**	**.43**	**.60**	-.01	**.71**	-
	(*n* = 28)	(*n* = 32)	(*n* = 36)	(*n* = 31)	(*n* = 35)	(*n* = 45)	
8.Monetary Probability Discounting Task	-.14	**.38**	**.38**	-.10	.18	.06	.02
	(*n* = 29)	(*n* = 32)	(*n* = 37)	(*n* = 31)	(*n* = 36)	(*n* = 46)	(*n* = 44)

*Note*: Bolded text indicates significance (*p* <. 05).

## Discussion

This study systematically examined discounting of delayed and probabilistic sexual and monetary outcomes among individuals with cocaine use disorders and demographically-matched controls. First, we found that individuals with cocaine use disorders discounted significantly more steeply than controls in two of the four Sexual Delay Discounting Task partner conditions, as well as in the Monetary Delay Discounting Task. Second, in the novel Sexual Probability Discounting Task, both groups showed an orderly effect in which odds against contracting an STI systematically decreased the likelihood of using an immediately available condom. Third, no robust group differences in probability discounting of sexual outcomes or monetary rewards were found. Finally, correlations showed sexual and monetary results were unrelated, for both delay and probability discounting tasks. Each finding will be discussed in turn.

To date, no other study has examined whether individuals with cocaine use disorders discount delayed condom-protected sex more than matched controls. After controlling for each individual’s likelihood of condom use when no delay was involved, individuals with cocaine use disorders discounted delayed condom-protected sex significantly steeper than controls in two of four partner conditions. We suspect that the relatively high rates of reported condom use in the “most likely to have an STI” partner condition were responsible for the inability to detect group differences. Although likelihood of condom use was relatively lower in the “least likely to have an STI” partner condition, a significant group difference was not obtained, perhaps due in part to similar ratings of condom use likelihood between groups at delays shorter than 1 day. There were no significant differences between individuals with cocaine use disorders and controls in likelihood of using immediately available condoms in any of the four Sexual Delay Discounting Task partner conditions, demonstrating that the between-group differences in likelihood of using delayed condoms were truly driven by differential responses to delay (i.e., delay discounting). Had this study examined only preferences about using immediately available condoms, an entire dimension of increased HIV risk behavior would have been overlooked. This highlights the importance of delay discounting as a contributor to the high rates of HIV risk behavior. For the Monetary Delay Discounting Task, the finding of steeper discounting in individuals with cocaine use disorders replicates several previous findings [18–21], contributing to overall confidence in our study findings.

In the novel Sexual Probability Discounting Task, decreased odds of contracting an STI systematically decreased the likelihood of using an immediately available condom in both groups, suggesting that perceived STI risk has a lawful effect on condom use regardless of cocaine use history. Probability discounting of sexual outcomes has been shown in college students using tasks assessing choices between certain shorter durations vs. uncertain longer durations of sexual activity [63–64] or choices between certain “less than ideal” and uncertain “ideal” sexual outcomes, with idealness represented visually by line length [65]. These tasks assessed a theoretically important issue, the effects of uncertainty of a sexual act on its value, while our task assessed the clinically-relevant effect of STI uncertainty on condom use. Orderly probability effects in all three tasks speak to the robust effect of probability on sexual outcomes, regardless of whether a probabilistic reward [63–65] or a probabilistic punishment (hypothetical STI contraction in the current study) is being assessed.

In contrast to the delay discounting results, no robust between-group differences in probability discounting of either sexual or monetary outcomes were observed. The only between-group difference detected was in the shape of the monetary probability discounting function; there was no reliable between-group difference across the range of different probabilities assessed. A prior study showed no association between drug use and probability discounting of sexual outcomes (although low drug use in the sample was a limitation [64]). Our comparisons between individuals with cocaine use disorders and matched controls regarding STI risk and condom use further suggest no robust relations between drug use and probability discounting of sexual outcomes. This conclusion is consistent with the larger literature on probability discounting, which shows mixed results regarding relations between drug use and probability discounting of monetary gains [66–72], and no apparent relation between drug use and probability discounting of monetary losses [69, 73]. Our findings therefore support the somewhat paradoxical conclusion that behavioral processes underlying risk-taking may be unrelated to drug use, and specifically cocaine use in the present study. Likewise, loss aversion (i.e., the tendency to overweight losses relative to equivalent gains [74]), or negativity bias [75] may be similarly unrelated to cocaine use disorders, given our observation that individuals with cocaine use disorders did not differ from matched controls in terms of their sensitivity to STI contraction risk (i.e., a loss), but did differ significantly in their sensitivity to delayed condom-protected sex (i.e., a gain). Collectively, our differential findings between delay and probability discounting highlight the importance of examining multiple behavioral processes in relation to clinically relevant behavior, and suggests that, with respect to sexual outcomes, steeper delay discounting of condom-protected sex, but not reduced sensitivity to STI probability, contributes to increased sexual HIV risk among individuals with cocaine use disorders.

Correlations showed sexual and monetary results were unrelated, for both delay and probability discounting. The sexual partner conditions were generally positively related within a single type of sexual task (delay or probability). Moreover, delay and probability discounting in the sexual tasks were generally positively related. These findings were in contrast to the lack of significant association between delay and probability discounting of monetary rewards, and in contrast to the lack of significant associations between the monetary and sexual results for either delay or probability discounting. The nonsignificant correlation (in the positive direction) between delay and probability discounting of money should be viewed in the context of mixed evidence on this relationship, with studies typically showing correlations in the positive direction but varying from weak to strong in correlation strength, and varying between significance and nonsignificance [40–41, 54, 76–77]. The predominantly nonsignificant correlations between discounting of delayed condom-protected sex and delayed money replicates previous findings [30, 32]. One potential explanation for the relation between the delay and probability sexual tasks is that choice behavior involving waiting for a delayed condom and avoidance of STI contraction recruit related processes. This seems especially plausible given the risk of STI contraction implicit in all sexual situations, including the “most/least want to have sex with” partner conditions in the Sexual Delay Discounting Task.

With respect to probability discounting, it should be noted that, unlike the delay discounting tasks, the probability tasks assess events of opposite valences, with the risk of contracting an STI or receiving $100 representing a probabilistic loss (punishment) and gain (reward), respectively. As a result of this difference, a general tendency toward risk-taking would be evidenced by steep discounting of STI risk and shallow discounting of the monetary reward (and vice versa for risk-aversion), resulting in a negative correlation between these measures [78]. The fact that we did not observe significant negative correlations between these measures lends some support to the conclusion that sexual outcomes are discounted uniquely relative to the discounting of monetary outcomes.

These results add to growing evidence [30–32, 64, 79] showing domain specificity in discounting results. In other words, discounting results differ depending upon the type of outcome studied. Most human discounting studies have examined only money as the outcome, with the implicit but questionable assumption that decision making for monetary outcomes is indicative of decision making in clinically relevant domains such as substance use disorders. Replicating previous findings with respect to opioid-dependent women [32], the present results suggest that delay discounting of condom-protected sex and delay discounting of money are different processes, although individuals with cocaine use disorders discount both more steeply than matched controls. Similarly, results suggest that probability discounting of STI contraction and probability discounting of money are different processes, but unlike with delay discounting, these different probability discounting processes do not differ between individuals with cocaine use disorders and matched controls who did not use cocaine.

One limitation is that despite matching participants demographically with respect to age, sex, race, ethnicity, marital status, education, monthly income, intelligence test score, and cigarettes per day, participants in the Cocaine group and the Control group differed on substance use variables other than cocaine use. Specifically, participants in the Cocaine group showed significantly higher rates of alcohol and cannabis use relative to participants in the Control group. Moreover, participants in the Cocaine group showed significantly higher self-reported sexual risk behavior and were significantly more likely to be HIV positive. We believe these differences are to be expected and indicate that we studied a representative sample of individuals with cocaine use disorders [2–6; 18–21]. Indeed, the higher rates of sexual risk behavior and HIV infection in the Cocaine group highlight the behavioral problems associated with cocaine use disorders, which prompted the present study. Further, there is some evidence to suggest that cocaine use may be a stronger predictor of discounting than alcohol and cannabis use. For example, individuals with problematic cocaine use discount more steeply than those with problematic alcohol use [21], and the relationship between cannabis use and discounting is less robust than previous studies with other drugs [51]. Finally, differences in other drug use between the Cocaine group and Control group are comparable to differences that may have been present in previous studies examining discounting in individuals who use cocaine relative to a matched control group [18–21].

An additional limitation is that the various discounting tasks involved hypothetical rather than real outcomes. However, delay and probability discounting studies have generally shown similar results when using real and hypothetical money ([20, 48–50, 80–86] but see [87–88]). Moreover, the Sexual Delay Discounting Task has demonstrated reliability and relationships with self-reported sexual risk [30–31, 33]. Another potential limitation is that discounting in the sexual tasks may have been affected not only by delay or probability, but also by the effort associated with condom use, whereas this was not the case in the monetary tasks. Although a potential effort-related confound could have contributed to the lack of significant correlations between the task types, attempts to control for this variable could jeopardize the external validity of the sexual tasks. Moreover, it is unlikely that effort influenced group differences in the Sexual Delay Discounting Task, because the Sexual Probability Discounting Task also involves the same potential effort-related confound, yet did not show robust group differences. Another shortcoming was our relatively small sample size. However, the orderliness of the data and the detection of between-groups differences suggest the sample was sufficient to detect meaningful results. Finally, although the present findings suggest an association with cocaine use disorders, the etiology of increased sexual HIV risk still remains unclear. To examine the potential contribution of cocaine pharmacology on sexual risk behavior, future research should examine the effects of acute cocaine administration on the Sexual Delay Discounting Task and the Sexual Probability Discounting Task in individuals who use cocaine.

The translational nature of this research contributes a novel perspective to the prevention of HIV sexual risk behavior among individuals with and without cocaine use disorders. Both delay and probability discounting may serve a diagnostic role, identifying individuals who are at risk for HIV or STI contraction (or transmission to others, as highlighted by the non-trivial percentage of our sample that was HIV-positive). Among individuals who exhibit high rates of sexual risk behavior and steeply discount delayed condom-protected sex, behavioral treatment strategies aimed at reinforcing condom carrying or training delay tolerance may reduce the likelihood of HIV and other STI transmission. Among individuals who exhibit high rates of sexual risk behavior and steeply discount the possibility of uncertain STI contraction, training that increases the perceived likelihood that partners may have an STI may also decrease HIV and other STI transmission. The present translational research on basic behavioral processes using clinically relevant decisions may therefore be leveraged to improve public health.

## References

[1] Substance Abuse and Mental Health Services Administration, Results from the 2013 National Survey on Drug Use and Health: Summary of National Findings, NSDUH Series H-48, HHS Publication No. (SMA) 14–4863. Rockville, MD: Substance Abuse and Mental Health Services Administration, 2014.

[2] Booth RE, Watters JK, Chitwood DD. HIV risk-related sex behaviors among injection drug users, crack smokers, and injection drug users who smoke crack. Am J Public Health. 1993; 83: 1144–1148. 8342724 10.2105/ajph.83.8.1144 PMC1695160

[3] Booth RE, Kwiatkowski CF, Chitwood DD. Sex related HIV risk behaviors: Differential risks among injection drug users, crack smokers, and injection drug users who smoke crack. Drug Alcohol Depend. 2000; 58: 219–226. 10759032 10.1016/s0376-8716(99)00094-0

[4] Mathers BM, Degenhardt L, Phillips B, Wiessing L, Hickman M, Strathdee SA, et al Global epidemiology of injecting drug use and HIV among people who inject drugs: A systematic review. Lancet. 2008; 372: 1733–1745. 10.1016/S0140-6736(08)61311-2 18817968

[5] McCoy CB, Lai S, Metsch LR, Messiah SE, Zhao W. Injection drug use and crack cocaine smoking: Independent and dual risk behaviors for HIV infection. Ann Epidemiology. 2004; 14: 535–542. 15350952 10.1016/j.annepidem.2003.10.001

[6] Strathdee SA, Sherman SG. The role of sexual transmission of HIV infection among injection and non-injection drug users. J Urban Health. 2003; 80: iii7–iii14. 14713667 10.1093/jurban/jtg078 PMC3456264

[7] Centers for Disease Control and Prevention: HIV and AIDS in the United States fact sheet, 2010 Department of Health and Human Services, Centers for Disease Control and Prevention http://www.cdc.gov/hiv/resources/factsheets/us.htm.

[8] Chaisson RE, Bacchetti P, Osmond D, Brodie B, Sande MA, Moss AR. Cocaine use and HIV infection in intravenous drug users in San Francisco. JAMA. 1989; 261: 561–565. 2909798

[9] Hudgins R, McCusker J, Stoddard A. Cocaine use and risky injection and sexual behaviors. Drug Alcohol Depend. 1995; 37: 7–14. 7882875 10.1016/0376-8716(94)01060-x

[10] Morissette C, Cox J, De P, Tremblay C, Roy É, Allard R, et al Minimal uptake of sterile drug preparation equipment in a predominantly cocaine injecting population: Implications for HIV and hepatitis C prevention. Int J Drug Policy. 2007; 18: 204–212. 17689367 10.1016/j.drugpo.2006.08.004

[11] Kral AH, Bluthenthal RN, Booth RE, Watters JK. HIV seroprevalence among street-recruited injection drug and crack cocaine users in 16 US municipalities. Am J Public Health. 1998; 88: 108–113. 9584014 10.2105/ajph.88.1.108 PMC1508387

[12] Heil SH, Sigmon SC, Mongeon JA, Higgins ST. Characterizing and improving HIV/AIDS knowledge among cocaine-dependent outpatients. Exp Clin Psychopharmacol. 2005; 13: 238–245. 16173887 10.1037/1064-1297.13.3.238

[13] Herrmann ES, Heil SH, Sigmon SC, Dunn KE, Washio Y, Higgins ST. Characterizing and improving HIV/AIDS knowledge among cocaine-dependent outpatients using modified materials. Drug Alcohol Depend. 2013; 127: 220–225. 10.1016/j.drugalcdep.2012.07.006 22889696 PMC4026286

[14] Winhusen TM, Somoza EC, Lewis DF, Kropp F, Theobald J, Elkashef A. An evaluation of substance abuse treatment and HIV education on safe sex practices in cocaine dependent individuals. ISRN Addiction. 2014; Article ID 912863. 10.1155/2014/912863 PMC4392976 25938124

[15] Malow RM, West JA, Corrigan SA, Pena JM, Cunningham SC. Outcome of psychoeducation for HIV risk reduction. AIDS Educ Prev. 1994; 6: 113–125. 8018438

[16] Prendergast ML, Urada D, Podus D. Meta-analysis of HIV risk-reduction interventions within drug abuse treatment programs. J Consult Clin Psychol. 2001; 69: 389–405. 11495169 10.1037//0022-006x.69.3.389

[17] Semaan S, Des Jarlais DC, Sogolow E, Johnson WD, Hedges LV, Ramirez G, et al A meta-analysis of the effect of HIV prevention interventions on the sex behaviors of drug users in the United States. J Acquir Immune Defic Syndr. 2002; 30: S73–93. 12107362

[18] Coffey SF, Gudleski GD, Saladin ME, Brady KT. Impulsivity and rapid discounting of delayed hypothetical rewards in cocaine-dependent individuals. Exp Clin Psychopharmacol. 2003; 11: 18–25. 12622340 10.1037//1064-1297.11.1.18

[19] Heil SH, Johnson MW, Higgins ST, Bickel WK. Delay discounting in currently using and currently abstinent cocaine-dependent outpatients and non-drug-using matched controls. Addict Behav. 2006; 31: 1290–1294. 16236455 10.1016/j.addbeh.2005.09.005

[20] Johnson MW. An efficient operant choice procedure for assessing delay discounting in humans: Initial validation in cocaine-dependent and control individuals. Exp Clin Psychopharmacol. 2012; 20: 191–204. 10.1037/a0027088 22329554 PMC3535463

[21] Kirby KN, Petry NM. Heroin and cocaine abusers have higher discount rates for delayed rewards than alcoholics or non-drug-using controls. Addiction. 2004; 99: 461–471. 15049746 10.1111/j.1360-0443.2003.00669.x

[22] MacKillop J, Amlung MT, Few LR, Ray LA, Sweet LH, Munafò MR. Delayed reward discounting and addictive behavior: A meta-analysis. Psychopharmacology. 2011; 216: 305–321. 10.1007/s00213-011-2229-0 21373791 PMC3201846

[23] Dixon MR, Marley J, Jacobs EA. Delay discounting by pathological gamblers. J Appl Behav Anal. 2003; 36: 449–458. 14768665 10.1901/jaba.2003.36-449 PMC1284461

[24] Petry NM. Pathological gamblers, with and without substance abuse disorders, discount delayed rewards at high rates. J Abnorm Psychol. 2001; 110: 482–487. 11502091 10.1037//0021-843x.110.3.482

[25] Weller RE, Cook EW, Avsar KB, Cox JE. Obese women show greater delay discounting than healthy-weight women. Appetite. 2008; 51: 563–569. 10.1016/j.appet.2008.04.010 18513828

[26] Fields SA, Sabet M, Reynolds B. Dimensions of impulsive behavior in obese, overweight, and healthy-weight adolescents. Appetite. 2013; 70: 60–66. 10.1016/j.appet.2013.06.089 23831015

[27] Axon RN, Bradford WD, Egan BM. The role of individual time preferences in health behaviors among hypertensive adults: A pilot study. J Am Soc Hypertens. 2009; 3: 35–41. 10.1016/j.jash.2008.08.005 20409943

[28] Bradford WD. The association between individual time preferences and health maintenance habits. Med Decis Making. 2010; 30: 99–112. 10.1177/0272989X09342276 19675322

[29] Daugherty JR, Brase GL. Taking time to be healthy: Predicting health behaviors with delay discounting and time perspective. Pers Indiv Differ. 2010; 48: 202–207.

[30] Johnson MW, Bruner NR. The Sexual Discounting Task: HIV risk behavior and the discounting of delayed sexual rewards in cocaine dependence. Drug Alcohol Depend. 2012; 123: 15–21. 10.1016/j.drugalcdep.2011.09.032 22055012 PMC3290676

[31] Johnson MW, Bruner NR. Test–retest reliability and gender differences in the sexual discounting task among cocaine-dependent individuals. Exp Clin Psychopharmacol. 2013; 21: 277–286. 10.1037/a0033071 23834552 PMC3880114

[32] Herrmann ES, Hand DJ, Johnson MW, Badger GJ, Heil SH. Examining delay discounting of condom-protected sex among opioid-dependent women and non-drug-using control women. Drug Alcohol Depend. 2014; 144: 53–60. 10.1016/j.drugalcdep.2014.07.026 25190049 PMC4252483

[33] Dariotis J, Johnson MW. Sexual discounting among high-risk urban youth ages 18–24: Implications for sexual and substance use risk behaviors. Exp Clin Psychopharmacol. 2015; 23: 49–58. 10.1037/a0038399 25545764 PMC4350924

[34] Lejuez CW, Bornovalova MA, Daughters SB, Curtin JJ. Differences in impulsivity and sexual risk behavior among inner-city crack/cocaine users and heroin users. Drug Alcohol Depend. 2005; 77: 169–175. 15664718 10.1016/j.drugalcdep.2004.08.013

[35] Bornovalova MA, Daughters SB, Hernandez GD, Richards JB., & Lejuez CW. Differences in impulsivity and risk-taking propensity between primary users of crack cocaine and primary users of heroin in a residential substance-use program. Exp Clin Psychopharmacol. 2005; 13: 311–318. 16366761 10.1037/1064-1297.13.4.311

[36] Bickel WK, Johnson MW, Koffarnus MN, MacKillop J, Murphy JG. The behavioral economics of substance use disorders: Reinforcement pathologies and their repair. Annu Rev Clin Psychol. 2014; 10: 641–677. 10.1146/annurev-clinpsy-032813-153724 24679180 PMC4501268

[37] Rachlin H, Raineri A, Cross D. Subjective probability and delay. J Exp Anal Behav. 1991; 55: 233–244. 2037827 10.1901/jeab.1991.55-233 PMC1323057

[38] Du W, Green L, Myerson J. Cross-cultural comparisons of discounting delayed and probabilistic rewards. Psychol Rec. 2002; 52: 479–492. 12420251

[39] Green L, Myerson J, Ostaszewski P. Amount of reward has opposite effects on the discounting of delayed and probabilistic outcomes. J Exp Psychol Learn. 1999; 25: 418–427. 10093208 10.1037//0278-7393.25.2.418

[40] Myerson J, Green L, Hanson JS, Holt DD, Estle SJ. Discounting delayed and probabilistic rewards: Processes and traits. J Econ Psychol. 2003; 24: 619–635.

[41] Holt DD, Green L, Myerson J. Is discounting impulsive?: Evidence from temporal and probability discounting in gambling and non-gambling college students. Behav Process. 2003; 64: 355–367. 14580704 10.1016/s0376-6357(03)00141-4

[42] Ammons RB, Ammons CH. The Quick Test (QT): provisional manual. Psychol Rep. 1962; 11: 111–161.

[43] Wilkinson GS, 1993 WRAT-3: Wide range achievement test administration manual Wilmington, DE: Wide Range.

[44] Hudziak JJ, Helzer JE, Wetzel MW, Kessel KB, McGee B, Janca A, Przybeck T. The use of the DSM-III-R Checklist for initial diagnostic assessments. Compr Psychiatry. 1993; 34: 375–383. 8131381 10.1016/0010-440x(93)90061-8

[45] Darke S, Hall W, Heather N, Ward J, Wodak A. The reliability and validity of a scale to measure HIV risk-taking behavior among intravenous drug users. AIDS. 1991; 5: 181–185. 2031690 10.1097/00002030-199102000-00008

[46] Petry N. Reliability of drug users’ self-reported HIV risk behaviors using a brief, 11-item scale. Subst Use Misuse. 2001; 36: 1731–1747. 11758820 10.1081/ja-100107576

[47] Centers for Disease Control and Prevention. Likely Female-to-Female Sexual Transmission of HIV. Morbidity and Mortality Weekly Report. 2014; 63: 209–210. http://www.cdc.gov/mmwr/pdf/wk/mm6310.pdf 24622284 PMC5779339

[48] Baker F, Johnson MW, Bickel WK. Delay discounting in current and never-before cigarette smokers: Similarities and differences across commodity, sign, and magnitude. J Abnorm Psychol. 2003; 112, 382–392. 12943017 10.1037/0021-843x.112.3.382

[49] Johnson MW, Bickel WK. Within-subject comparison of real and hypothetical money rewards in delay discounting. J Exp Anal Behav. 2002; 77: 129–146. 11936247 10.1901/jeab.2002.77-129 PMC1284852

[50] Johnson MW, Bickel WK, Baker F. Moderate drug use and delay discounting: A comparison of heavy, light, and never smokers. Exp Clin Psychopharmacol. 2007; 15: 187–194. 17469942 10.1037/1064-1297.15.2.187

[51] Johnson MW, Bickel WK, Baker F, Moore BA, Badger GJ, Budney AJ. Delay discounting in current and former marijuana-dependent individuals. Exp Clin Psychopharmacol. 2010; 18: 99–107. 10.1037/a0018333 20158299 PMC2874198

[52] Yi R, Johnson MW, Bickel WK. Relationship between cooperation in an iterated prisoner's dilemma game and the discounting of hypothetical outcomes. Learn Behav. 2005; 33: 324–336. 16396079 10.3758/bf03192861

[53] Yoon JH, Higgins ST, Heil SH, Sugarbaker RJ, Thomas CS, Badger GJ. Delay discounting predicts postpartum relapse to cigarette smoking among pregnant women. Exp Clin Psychopharmacol. 2007; 15: 176–186. 17469941 10.1037/1064-1297.15.2.186

[54] Richards JB, Zhang L, Mitchell SH, Wit H. Delay or probability discounting in a model of impulsive behavior: effect of alcohol. J Exp Anal Behav. 1999; 71: 121–143. 10220927 10.1901/jeab.1999.71-121 PMC1284697

[55] Kaplan BA, Reed DD, McKerchar TL. Using a visual analogue scale to assess delay, social, and probability discounting of an environmental loss. Psychol Rec. 2014; 64: 261–269.

[56] Johnson MW, Bickel WK. An algorithm for identifying nonsystematic delay-discounting data. Exp Clin Psychopharmacol. 2008; 16: 264–274. 10.1037/1064-1297.16.3.264 18540786 PMC2765051

[57] Johnson PS, Herrmann ES, Johnson MW. Opportunity costs of reward delays and the discounting of hypothetical money and cigarettes. J Exp Anal Behav. 2015; 103: 87–107. 10.1002/jeab.110 25388973 PMC4428151

[58] Motulsky HJ, Christopoulos A. Fitting models to biological data using linear and nonlinear regression: A practical guide to curve fitting San Diego, CA: GraphPad Software, Inc.; 2003.

[59] Green L, Fry AF, Myerson J. Discounting of delayed rewards: A life-span comparison. Psychol Sci. 1994; 5, 33–36.

[60] Myerson J, Green L. Discounting of delayed rewards: Models of individual choice. J Exp Anal Behav. 1995; 64: 263–276. 16812772 10.1901/jeab.1995.64-263 PMC1350137

[61] Rachlin H. Judgment, decision, and choice: A cognitive/behavioral synthesis WH Freeman/Times Books/Henry Holt Co.; 1989.

[62] Myerson J, Green L, Warusawitharana M. Area under the curve as a measure of discounting. J Exp Anal Behav. 2001; 76: 235–243. 11599641 10.1901/jeab.2001.76-235 PMC1284836

[63] Lawyer SR, Williams SA, Prihodova T, Rollins JD, Lester AC. Probability and delay discounting of hypothetical sexual outcomes. Behav Process. 2010; 84: 687–692. 10.1016/j.beproc.2010.04.002 20385215

[64] Lawyer SR, Schoepflin FJ. Predicting domain-specific outcomes using delay and probability discounting for sexual versus monetary outcomes. Behav Process. 2013; 96: 71–78. 10.1016/j.beproc.2013.03.001 23500484

[65] Holt DD, Newquist MH, Smits RR, Tiry AM. Discounting of food, sex, and money. Psychon Bull Rev. 2014; 21: 794–802. 10.3758/s13423-013-0557-2 24338570

[66] Andrade LF, Petry NM. Delay and probability discounting in pathological gamblers with and without a history of substance use problems. Psychopharmacology. 2012; 219: 491–499. 10.1007/s00213-011-2508-9 21952671 PMC3629698

[67] Reynolds B, Karraker K, Horn K, Richards JB. Delay and probability discounting as related to different stages of adolescent smoking and non-smoking. Behav Process. 2003; 64: 333–344. 10.1016/s0376-6357(03)00168-2 14580702

[68] Reynolds B, Richards JB, Horn K, Karraker K. Delay discounting and probability discounting as related to cigarette smoking status in adults. Behav Process. 2004; 65: 35–42. 10.1016/s0376-6357(03)00109-8 14744545

[69] Takahashi T, Ohmura Y, Oono H, Radford M. Alcohol use and discounting of delayed and probabilistic gain and loss. Neuroendocrinol Lett. 2009; 30: 749–752. 20038936

[70] Yi R, Landes RD. Temporal and probability discounting by cigarette smokers following acute smoking abstinence. Nicotine Tob Res. 2012; 14: 547–558. 10.1093/ntr/ntr252 22311959 PMC3337536

[71] Yi R, Chase WD, Bickel WK. Probability discounting among cigarette smokers and nonsmokers: Molecular analysis discerns group differences. Behav Pharmacol. 2007; 18: 633–639. 17912047 10.1097/FBP.0b013e3282effbd3

[72] Yi R, Carter AE, Landes RD. Restricted psychological horizon in active methamphetamine users: Future, past, probability, and social discounting. Behav Pharmacol. 2012; 23: 358–366. 10.1097/FBP.0b013e3283564e11 22743602 PMC4104478

[73] Ohmura Y, Takahashi T, Kitamura N. Discounting delayed and probabilistic monetary gains and losses by smokers of cigarettes. Psychopharmacology. 2005; 182: 508–515. 16167142 10.1007/s00213-005-0110-8

[74] Kahneman D, Tversky A. Choices, values, and frames. Am Psychol. 1984; 39: 341–350.

[75] Baumeister RF, Finkenauer C, Vohs KD. Bad is stronger than good. Rev Gen Psychol. 2001; 5: 323–370.

[76] Mitchell SH. Measures of impulsivity in cigarette smokers and non-smokers. Psychopharmacology. 1999; 146: 455–464. 10550496 10.1007/pl00005491

[77] Jarmolowicz DP, Bickel WK, Carter AE, Franck CT, Mueller ET. Using crowdsourcing to examine relations between delay and probability discounting. Behav Process. 2012; 91: 308–312. 10.1016/j.beproc.2012.09.001 22982370 PMC4732266

[78] Shead NW, Hodgins DC. Probability discounting of gains and losses: Implications for risk attitudes and impulsivity. J Exp Anal Behav. 2009; 92: 1–16. 10.1901/jeab.2009.92-1 20119519 PMC2707142

[79] Rasmussen EB, Lawyer SR, Reilly W. Percent body fat is related to delay and probability discounting for food in humans. Behav Process. 2010; 83: 23–30. 10.1016/j.beproc.2009.09.001 19744547

[80] Bickel WK, Pitcock JA, Yi R, Angtuaco EJ. Congruence of BOLD response across intertemporal choice conditions: Fictive and real money gains and losses. J Neurosci. 2009; 29: 8839–8846. 10.1523/JNEUROSCI.5319-08.2009 19587291 PMC2749994

[81] Green RM, Lawyer SR. Steeper delay and probability discounting of potentially real versus hypothetical cigarettes (but not money) among smokers. Behav Process. 2014; 108: 50–56. 10.1016/j.beproc.2014.09.008 25225037

[82] Lagorio CH, Madden GJ. Delay discounting of real and hypothetical rewards III: Steady-state assessments, forced-choice trials, and all real rewards. Behav Process. 2005; 69: 173–187. 10.1016/j.beproc.2005.02.003 15845306

[83] Lawyer SR, Schoepflin F, Green R, Jenks C. Discounting of hypothetical and potentially real outcomes in nicotine-dependent and nondependent samples. Exp Clin Psychopharmacol. 2011; 19: 263–274. 10.1037/a0024141 21707190

[84] Madden GJ, Begotka AM, Raiff BR, Kastern LL. Delay discounting of real and hypothetical rewards. Exp Clin Psychopharmacol. 2003; 11: 139–145. 12755458 10.1037/1064-1297.11.2.139

[85] Madden GJ, Raiff BR, Lagorio CH, Begotka AM, Mueller AM, Hehli DJ, et al Delay discounting of potentially real and hypothetical rewards: II. Between-and within-subject comparisons. Exp Clin Psychopharmacol. 2004; 12: 251–261. 15571442 10.1037/1064-1297.12.4.251

[86] Matusiewicz AK, Carter AE, Landes RD, Yi R. Statistical equivalence and test–retest reliability of delay and probability discounting using real and hypothetical rewards. Behav Process. 2013; 100: 116–122. 10.1016/j.beproc.2013.07.019 PMC4116793 23954833

[87] Hinvest NS, Anderson IM. The effects of real versus hypothetical reward on delay and probability discounting. Q J Exp Psychol. 2010; 63: 1072–1084. 10.1080/17470210903276350 19890767

[88] Jikko Y, Okouchi H. Real and hypothetical rewards in probability discounting. The Japanese Journal of Psychology. 2007; 78: 269–276. 17892024 10.4992/jjpsy.78.269

